# Cutaneous Melanoma in a Rabbit With Multiple Metastatic Lesions: A Case Report

**DOI:** 10.1155/crve/8573115

**Published:** 2026-04-28

**Authors:** Amir Rostami, Hossein Aminianfar, Niloofar Zarifian, Nazanin Samiee, Ali Rahmanifar, Tarane Behniapour

**Affiliations:** ^1^ Department of Internal Medicine, Faculty of Veterinary Medicine, University of Tehran, Tehran, Iran, ut.ac.ir; ^2^ Department of Pathology, Faculty of Veterinary Medicine, University of Tehran, Tehran, Iran, ut.ac.ir; ^3^ Faculty of Veterinary Medicine, University of Tehran, Tehran, Iran, ut.ac.ir

**Keywords:** cutaneous neoplasm, immunohistochemistry, melanoma, metastasis, *Oryctolagus cuniculus*, rabbit

## Abstract

**Background:**

Neoplasia is increasingly common in senior pet rabbits, with cutaneous melanoma being a rare but aggressive type. Its characteristics in rabbits are not fully understood.

**Case Description:**

A 5‐year‐old rabbit initially presented with a cutaneous mass at the ear base. Despite surgical excision, it recurred rapidly with multiple facial/chest masses and pulmonary metastases, leading to euthanasia. Pathological evaluation confirmed malignant melanoma, revealing features such as high mitotic activity and lymphovascular invasion; immunohistochemistry provided the definitive diagnosis.

**Conclusion and Clinical Relevance:**

This case highlights the highly aggressive and metastatic nature of cutaneous melanoma in rabbits, often resulting in a poor prognosis. Clinicians should be aware of melanoma’s aggressive potential in rabbits. Surgical intervention alone may prove inadequate, and current treatment options in rabbits are limited. In this case, surgical intervention was not effective, likely because micrometastasis was already present. Treatment options remain limited, and euthanasia is often required in metastatic cases.

## 1. Introduction

As pet rabbits (*Oryctolagus cuniculus*) increasingly live to their full potential lifespan, there has been a corresponding rise in the diagnosis of neoplastic diseases [1–3]. The incidence of neoplasms in rabbits rises directly with age, affecting up to 47.2% of the population that is older than 6 years [2]. Recent retrospective studies have documented a higher prevalence of spontaneous neoplasms in pet rabbits compared to laboratory rabbits, where a prevalence rate of 2.7% is observed in animals over 2 years old [1–5].

Histopathological analysis of tissues from pet rabbits reveals a high prevalence of skin cancer, accounting for 47% of submitted specimens. While most cutaneous epithelial tumors are benign, those of mesenchymal origin are frequently diagnosed as malignant [1, 3]. Myxosarcoma, fibroma, fibrosarcoma, papilloma, basal cell carcinoma, squamous cell carcinoma, sebaceous gland carcinoma, and lymphoma are among the skin tumor types reported in rabbits, with trichoblastoma consistently reported as the single most common skin tumor type found in pet rabbits [1–3, 6].

Cutaneous melanoma is relatively rare in rabbits, with its incidence reported at 4.2% among cutaneous tumors [3]. It is hypothesized that UV light contributes to the development of melanocytic tumors in rabbits, given their common appearance on parts of the head with minimal hair coverage, such as the eyelid and ear pinna [1, 7]. A hallmark of rabbit melanomas is their aggressive nature, frequently demonstrated by their tendency to metastasize to the lymph nodes, lungs, liver, spleen, and other organs, with lymphovascular invasion [5, 8, 9].

Due to the limited availability of documented cases, critical components of rabbit melanoma, including its usual anatomical distribution, histopathological characteristics, immunophenotypic profile, and prognostic indicators, remain inadequately delineated. The present case report is aimed at contributing significant data to this restricted body of knowledge by offering a thorough examination, from clinical presentation and macroscopic pathology to histopathological analysis and immunohistochemical profiling, of a pet rabbit diagnosed with metastatic cutaneous melanoma.

## 2. Case Description

A 5‐year‐old, entire, male mixed‐breed rabbit was presented with a history of dental complications requiring veterinary intervention every 4–5 weeks for incisor teeth trimming. The rabbit’s diet consisted primarily of alfalfa hay, supplemented with commercial pellets and a variety of fruits. The first cutaneous nodule at the base of the left ear was detected when the rabbit was approximately 3 years old. About 14 months later, the mass was surgically excised at another clinic during a scheduled visit for routine dental trimming. The mass was described as a solitary, darkly pigmented cutaneous nodule located at the base of the ear; however, no cytological or histopathological data from that procedure were available. Within a few months, multiple neoplastic lesions of varying sizes emerged on the rabbit’s facial and chest skin, and the rabbit was subsequently referred to our hospital. At presentation, the rabbit was in poor general condition, with noticeable weight loss, marked lethargy, and reduced activity, likely exacerbated by chronic dental disease. The patient was not receiving any medications at the time of referral. Given the rapid disease progression, extensive tissue involvement, concern for internal organ compromise, and deterioration of the patient’s condition, no complementary exam was performed, and humane euthanasia was elected.

### 2.1. Pathological Examination

After euthanasia, a systematic necropsy was performed, and tissue specimens from cutaneous masses and affected metastatic foci of the lung were harvested and submitted to the histopathology laboratory, Department of Veterinary Pathology, University of Tehran, Iran. Tissue specimens were fixed with neutral buffered formalin solution 10% for 48 h, routinely processed for histology, embedded in paraffin wax, and sectioned at a 5‐*μ*m thickness. Sections were stained with hematoxylin and eosin (H&E) for routine histopathological evaluation.

### 2.2. Immunohistochemical Investigations

Immunohistochemistry (IHC) was performed to confirm the melanocytic origin. IHC was performed on 3‐*μ*m‐thick FFPE tissue sections. After deparaffinization and rehydration, heat‐induced epitope retrieval was conducted using citrate buffer (pH 6.0) in an incubator for 30 min. Endogenous peroxidase activity was quenched with 3% hydrogen peroxide for 10 min. Sections were incubated with a primary anti‐S100 antibody (1:100; monoclonal anti‐mouse, AB136629, Abcam) and then with Melan‐A (1:100; MART‐1 monoclonal anti‐mouse, A00133, ScyTek) for 1 h at room temperature. Immunoreactivity was visualized using 3,3 ^′^‐diaminobenzidine (DAB) chromogen (Dako). Sections were counterstained with Mayer’s hematoxylin, dehydrated, and coverslipped. Appropriate negative controls were included in which primary antibodies were replaced with nonimmune serum to exclude false positive results.

## 3. Results

### 3.1. Gross Pathology

**Figure 1:**
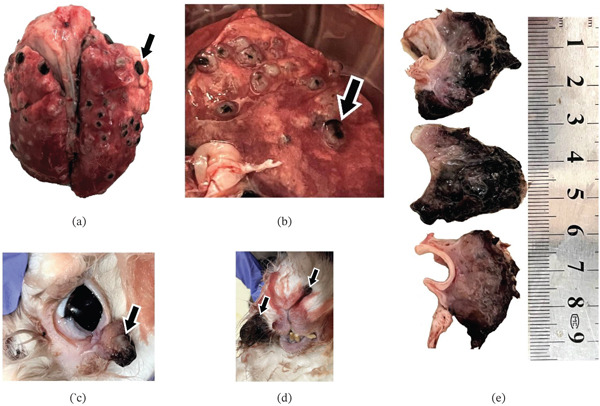
Gross pathology of primary cutaneous melanoma in a rabbit. (a, b) Gross pathology of pulmonary metastases in a rabbit with cutaneous melanoma. Multiple discrete, black metastatic nodules are scattered throughout the pulmonary parenchyma, indicated by black arrows. (c–e) Gross pathology of primary cutaneous masses. Lesions on the facial (nostril, palpebra, and cheek) and chest skin, measuring approximately each, appeared as raised, black, partially ulcerated masses with irregular surfaces, indicated by black arrows.

At necropsy, primary cutaneous masses were identified on the facial (nostril, palpebra, and cheek) and chest skin, measuring approximately 1.5 × 2 cm each. The neoplasm was heavily pigmented, appearing dark brown to black, with an irregular surface characterized by extensive central skin lesion ulceration. The mass had a firm consistency and infiltrative margins extending into the underlying dermal tissues. Examination of the respiratory system revealed multiple well‐demarcated, spherical, black nodules scattered throughout all lung lobes, ranging from 0.3 to 0.6 cm in diameter (Figure 1).

### 3.2. Histopathological Findings

**Figure 2:**
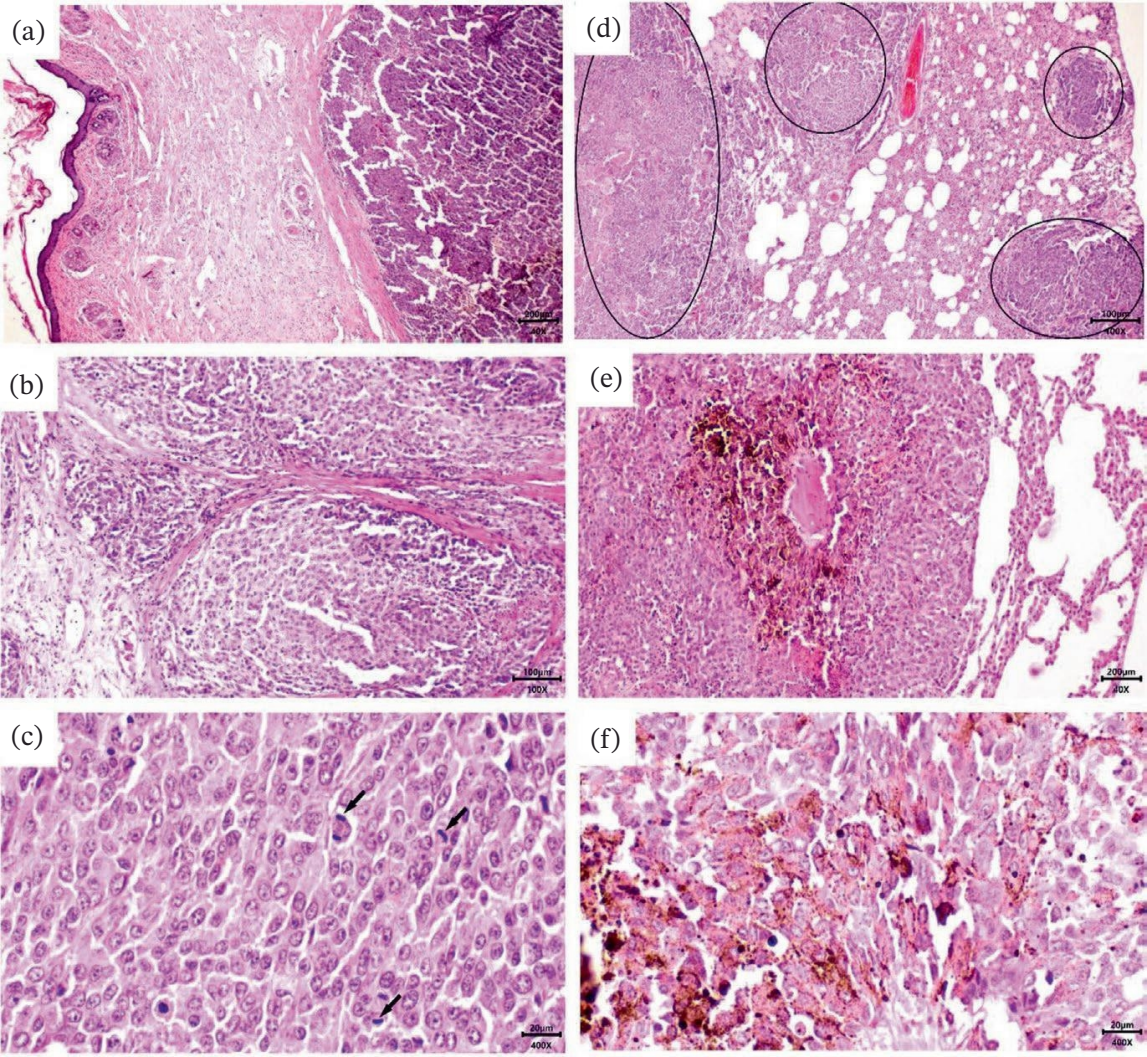
Primary cutaneous melanoma and its pulmonary metastatic lesions. (a) A densely cellular, asymmetrical neoplastic proliferation within the dermis with a normal skin margin (H&E staining, 40X). (b) Infiltrative growth pattern with neoplastic melanocytes arranged in nests and sheets. Prominent desmoplastic stromal response is evident with collagen bundle disruption (H&E staining, 100X). (c) Marked cellular pleomorphism with epithelioid and spindle cell morphologies. Neoplastic melanocytes display prominent nucleoli, nuclear atypia, and mitotic figures (arrows) (H&E staining, 400X). (d) Pulmonary tissue demonstrating multiple metastatic melanoma nodules (circled) distributed throughout the lung parenchyma (H&E staining, 40X). (e) Pulmonary metastatic focus showing densely packed neoplastic melanocytes with notable melanin pigmentation (brown granular material). The metastatic lesion exhibits central necrosis and hemorrhage, with compression of adjacent alveolar spaces (H&E staining, 100X). (f) Pulmonary metastasis demonstrating marked pleomorphism, prominent nucleoli, and abundant intracytoplasmic melanin pigmentation (H&E staining, 400X).

Histopathological evaluation of the primary cutaneous lesion demonstrated an infiltrative, nonencapsulated neoplastic mass extending from the epidermal layer into the deep dermis. The neoplasm comprised densely packed epithelioid to spindle‐shaped melanocytes, exhibiting a pattern of nests and sheets, with marked cellular pleomorphism and nuclear atypia with prominent nucleoli. The mitotic count was 4–6 per 2.37 mm^2^, and evidence of lymphovascular invasion was observed. Examination of the pulmonary parenchyma revealed multiple discrete, well‐demarcated neoplastic foci, consisting of densely cellular aggregates of pleomorphic melanocytes with variable melanin pigmentation. Areas of central necrosis and hemorrhage were evident within larger metastatic nodules (Figure 2).

**Figure 3:**
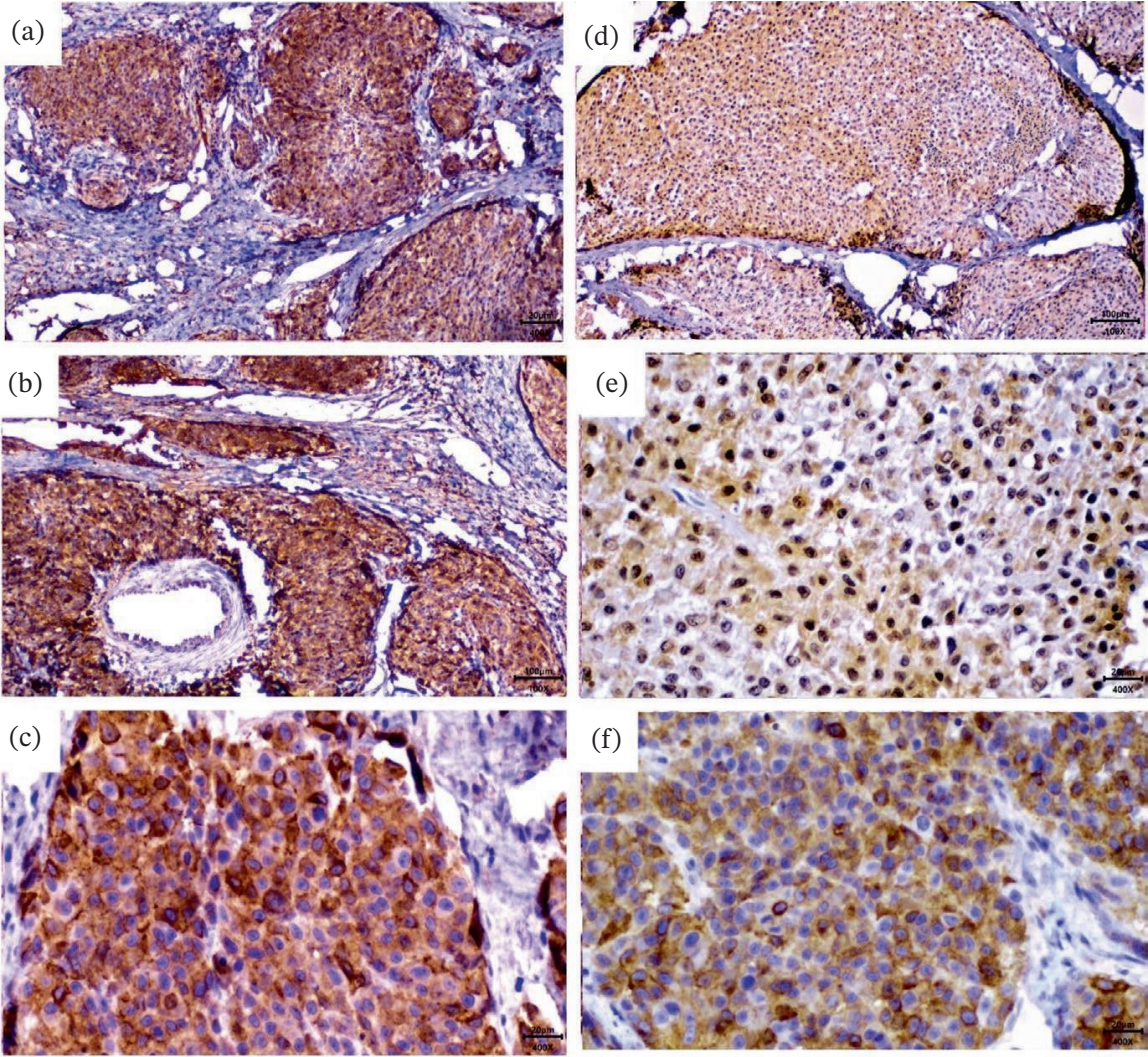
Immunohistochemical profile performed for the skin tumor and its pulmonary metastatic lesions. (a–c) Immunohistochemical staining for Melan‐A (MART‐1) using DAB chromogen (brown) with hematoxylin counterstain (blue purple) demonstrating strong cytoplasmic positivity in neoplastic melanocytes (a, 40X; b, 100X; c, 400X). (d–f) S100 protein immunohistochemical staining of pulmonary metastatic melanoma demonstrating both nuclear and cytoplasmic immunoreactivity that confirms the melanocytic origin of the metastatic tumor cells at low‐power (d, 40X), medium‐power (e, 100X), and high‐power (f, 400X) magnifications.

Immunohistochemical characterization confirmed the melanocytic origin in both primary and metastatic pulmonary lesions. The primary cutaneous tumor exhibited strong Melan‐A (MART‐1) and S100 immunoreactivity in the neoplastic cells. The dual positivity for Melan‐A and S100 protein provides definitive evidence of the melanocytic lineage of this neoplasm (Figure 3).

## 4. Discussion

This case report describes a cutaneous malignant melanoma with multiple metastatic lesions in a rabbit, contributing to the limited literature on spontaneous melanomas in this species. A comprehensive review identified cutaneous melanoma as a relatively infrequent finding, accounting for only 13 of the 249 primary skin neoplasms diagnosed in pet rabbits. These masses were commonly found in specific anatomical sites, including the skin of the dorsal trunk, perianal region, pinna, and eyelid. The rabbits diagnosed with cutaneous melanoma had a mean age of approximately 62.7 months [2], which is consistent with our case (5 years old, approximately 60 months).

The melanoma in this case exhibited several features consistent with high malignancy, including rapid growth, high mitotic index, prominent cellular pleomorphism, lymphovascular invasion, and metastasis. These findings align with previous reports suggesting that cutaneous melanomas in rabbits are typically aggressive with a poor prognosis [3,5,9].

The anatomic location of the primary tumor at the base of the ear has previously been associated with increased UV exposure in sparsely haired regions. However, this rabbit lived exclusively indoors without direct sunlight exposure, making UV radiation an unlikely contributing factor in this case [1, 7]. The etiology of melanoma remains incompletely elucidated. In rabbits, there are no definitive breed, sex, or age‐related predispositions reported, although older males seem to be more affected, with the New Zealand white breed appearing to be overrepresented [9, 10]. Conversely, specific risk factors have been identified in other species. In humans, risk factors include race, hypopigmentation, excessive solar exposure, and existing nevi [11].

Despite the small number of documented cases of malignant melanoma in rabbits, common pathological features include lymphovascular invasion, distant metastases, and tumor recurrence [9]. Lung metastases are common and have been identified in multiple rabbit and ferret cases with different primary sites, often extensively replacing lung tissue. In addition to the lungs, multiorgan metastasis was observed, including the liver, spleen, kidneys, lymph nodes (submandibular and popliteal), pleura/diaphragm, heart, adrenal glands, pituitary gland, trachea, stomach, aortic adventitia, skeletal muscle, and, notably, widespread bone marrow involvement in one case originating from a digit. Testicular metastasis from a scrotal primary melanoma was also documented, an unusual site previously described mainly in humans [5, 8, 9]. In our case, metastases were identified in the lungs, a common site for melanoma metastasis across species, and this pulmonary tropism appears characteristic of rabbit melanoma.

Although the anatomic location of melanoma is not entirely the sole determinant, it is a strong predictor of both local invasiveness and likelihood of metastasis, as in oral and/or mucosal melanomas, in which the highest degree of invasiveness and metastatic potential is expected, whereas tumors that develop on the limbs are usually associated with reduced malignancy [12,13].

In this case, despite the cutaneous origin at the base of the ear, the tumor exhibited aggressive behavior with rapid recurrence after surgical excision and development of pulmonary metastases.

The use of immunohistochemistry to identify specific melanocyte markers—such as Melan‐A (MART‐1), HMB‐45, S100, microphthalmia‐associated transcription factor, tyrosinase, neuron‐specific enolase, and PNL2—has become a standard diagnostic approach for melanoma in human, canine, and feline patients [2, 3, 8, 9, 14]. The use of multiple markers is essential for enhancing diagnostic accuracy, as demonstrated in this case, in which two melanocytic markers (Melan‐A and S100) were used to confirm the diagnosis. Studies suggest that IHC can also serve as a prognostic indicator; the ratio of Ki‐67‐positive cells may correlate directly with the prognosis of cutaneous melanoma in rabbits [8].

The occurrence of melanoma in dogs has shown genetic parallels to human melanoma. In dogs, mutations in NRAS, TP53, PTEN, KIT, KRAS, and NF1 have been found in oral melanomas, reflecting genetic alterations also discovered in human mucosal melanomas [7]. Genetic relations with spontaneous melanoma in rabbits have not yet been investigated and need to be addressed. Such data offer valuable insights into human melanomas and can develop new therapeutic options and diagnostic evaluations.

Treatment options for cancer in rabbits remain limited. For melanoma, the primary treatments currently include photodynamic therapy (PDT), cryotherapy, laser therapy, radiotherapy, or surgical excision [15]. Success rates for chemotherapy protocols in rabbits vary considerably and may be associated with significant side effects, including gastrointestinal stasis, anemia, inappetence, and exacerbation of subclinical infections [5, 14, 16].

## 5. Conclusion

The clinical course in this rabbit illustrates that surgical excision, while often the primary treatment for cutaneous tumors, may not prevent progression of biologically aggressive melanomas in some cases. Despite surgery, the disease advanced rapidly, with multiple new cutaneous masses and pulmonary metastases developing within months. This underscores the significance of early histopathological examination, meticulous follow‐up subsequent to any mass excision, and candid communication with owners regarding prognosis. Given the limited therapeutic alternatives accessible for rabbits, comprehensive documentation of similar cases remains essential for enhancing clinical comprehension and refining management strategies.

## Funding

No funding was received for this manuscript.

## Consent

The authors have nothing to report.

## Conflicts of Interest

The authors declare no conflicts of interest.

## Data Availability

The data that support the findings of this study are available from the corresponding authors upon reasonable request.
